# Evaluation of the Association Between Sedentary Time and Low Work Engagement in the Work Environment After COVID-19 Pandemic: A Cross-Sectional Study of Japanese Workers

**DOI:** 10.7759/cureus.62725

**Published:** 2024-06-19

**Authors:** Motoi Miura, Takahiro Tabuchi, Hoichi Amano, Kota Katanoda

**Affiliations:** 1 Epidemiology and Public Health, Tokyo Higaeri Day Surgery Clinic, Tokyo, JPN; 2 Cancer Control Center, Osaka International Cancer Institute, Osaka, JPN; 3 Public Health, The Tokyo Foundation for Policy Research, Tokyo, JPN; 4 Public Health, Teikyo University Graduate School of Public Health, Tokyo, JPN; 5 Division of Population Data Science, National Cancer Center Institute for Cancer Control, Tokyo, JPN

**Keywords:** work from home, desk worker, work engagement, covid-19, sedentary behavior

## Abstract

Introduction

The global shift toward working from home due to the COVID-19 pandemic has led to concerns about increased sedentary behavior and its potential impact on work engagement, a critical factor for employee well-being and organizational productivity. This study aims to explore the association between sedentary time and work engagement among workers in Japan in the post-pandemic work environment.

Methods

This cross-sectional analysis utilized data from the Japan COVID-19 and Society Internet Survey (JACSIS), conducted from September to November 2023, after the COVID-19 pandemic period. Participants included employed individuals over 18 years, excluding those in domestic occupations. Sedentary time and work engagement were self-reported and categorized. Logistic regression analysis adjusted for confounders such as socioeconomic status, work characteristics, and mental and physical health was employed to explore this association.

Results

The study found a significant association between longer sedentary time and lower levels of work engagement. In particular, for desk workers, longer sedentary time was associated with lower work engagement (sedentary time, compared to the reference category “<4 hours/day”, 4 to <8 h: OR 1.42, 95% CI: 1.25-1.60; 8 to <12 h: OR 1.77, 95% CI: 1.55-2.01; ≥12 h or unknown: OR 2.14, 95% CI: 1.80-2.51, respectively). Sensitivity analysis confirmed that these results are robust to different definitions of work engagement. Furthermore, analyses in subgroups of desk workers classified according to specific characteristics suggested that desk workers who are full-time workers in non-managerial positions and work from home ≥4 days per week were more strongly associated with prolonged sedentary behavior and low work engagement (in the group of full-time workers who were non-managers, sedentary time, compared to the reference category “<4 hours/day”, 4 to <8 h: OR 2.14, 95% CI: 1.52-3.00; 8 to <12 h: OR 2.10, 95% CI: 1.46-3.00; ≥12 h or unknown: OR 3.32, 95% CI: 1.99-6.05; in those with work-from-home frequency of ≥4 days weekly, sedentary time, compared to the reference category “<4 hours/day”, 4 to <8 h: OR 1.46, 95% CI: 0.99-2.16; 8 to <12 h: OR 1.73, 95% CI: 1.19-2.56; ≥12 h or unknown: OR 2.41, 95% CI: 1.58-3.67).

Conclusions

This study revealed a significant association between sedentary time and low work engagement among workers in Japan after the COVID-19 pandemic. In the future, prospective studies are needed to confirm the causal associations between the two, using more validated measures of sedentary behavior.

## Introduction

Work engagement is a measurement of a positive attitude toward work and comprises three components: vigor, deduction, and absorption [1,2]. People with high work engagement are less likely to experience burnout and have a lower risk of mental illness [3,4]. In addition, work engagement may be related to work-related behaviors such as sedentary behavior [5-7]. For example, a study of a 1966 birth cohort suggested a significant association between sedentary time and work engagement scores [5]. In a study involving Japanese individuals aged 20-59 years, the risk of low work engagement was 1.49 times higher in the group with longer sedentary time [6]. Another cross-sectional study suggested a significant association between sedentary time and work engagement among white-collar workers, even after adjusting for the effects of occupation [7].

However, these studies have several limitations. One is that most previous studies were conducted before the COVID-19 pandemic. Owing to the COVID-19 pandemic, working from home has become more common. This has resulted in increased sedentary time and decreased work engagement, which may have affected the nature and strength of the association between sedentary behavior and work engagement [8,9]. Moreover, although one of the aforementioned studies was conducted during the pandemic, the study participants answered the questions during Japan’s state of emergency, which has implications similar to the lockdown [7]. Work engagement may have been underestimated during Japan’s state of emergency compared to other periods during the pandemic, as access to entertainment facilities and restaurants was restricted and people were asked to refrain from going out. These restrictions may have worsened their mental state and reduced work engagement [10,11].

While behavioral restrictions will cease once the COVID-19 pandemic is controlled, work from home will become more common, and few studies, to our knowledge, have investigated whether sedentary time is associated with work engagement under such circumstances. To address this knowledge gap, this study aimed to investigate the association between sedentary time and work engagement in a society wherein working from home is becoming more common.

## Materials and methods

This cross-sectional study used data from the Japan COVID-19 and Society Internet Survey (JACSIS). The survey began on September 25, 2023, approximately two years after Japan’s last state of emergency, and ended on November 17, 2023, with 33,000 respondents [12]. This study was conducted after review and approval by the Ethics Review Committee of the Osaka International Cancer Institute (No. 20084-6) and the Research Ethics Review Committee of the National Cancer Center (No. 2020-447). 

The study population was defined as those who were ≥18 years old and employed in some type of occupation, excluding domestic work. Continuous variables considered outliers were excluded from the study population. Outliers were defined as values ≥third quartile plus 1.5 times the quartile range, or ≤first quartile minus 1.5 times the quartile range, in accordance with previous literature [13].

The exposure variable was self-reported sedentary time to the question which is similar to the question that examines sedentary time in the International Physical Activity Questionnaire (IPAQ), "How long have you been sitting on average in the last month?" with respondents choosing from 12 options (0 h to ≥12 h) [14]. In the analysis, sedentary time was classified into four categories based on a previous study (<4 h, 4 to <8 h, 8 to <12 h, and ≥12 h or unknown) [15]. The outcome variable was the degree of work engagement. Specifically, in accordance with previous research, participants were asked to select their responses (yes, somewhat yes, somewhat no, or no) to the question of whether they felt energized at work in the most recent month [9].

Both exposure and outcome variables were collected between September 25, 2023, and November 17, 2023. Although the outcome variable, work engagement, is thought to be influenced by the COVID-19 pandemic status, it was measured only once in this study. However, we believe that the work engagement data used in the analysis are appropriate for the point in time when the COVID-19 pandemic status has settled. In fact, no state of emergency has been issued in any region of Japan since October 1, 2021, and the legal classification of COVID-19 has been the same as that of seasonal influenza since May 8, 2023, indicating that the infection situation was stable at the time the data used in the analysis were obtained [12,16].

We employed several control variables to eliminate confounding factors. Specifically, socioeconomic variables (sex, age, body mass index, most recent educational background, and annual household income) were employed. In addition, we employed potential confounding factors as control variables. The inclusion criteria for the control variables were defined as factors that had an effect on either exposure or outcome based on a previous study [17]. The following variables were employed, based on the results of previous studies [18-25]. For each variable, several categories were created and dummy variables were assigned in order from 0 to the next before they were used in the analysis.

1. Labor time per week (0: <40 h or 1: ≧40 h)

2. Frequency of working from home (0: 0-3 times per month, 1: 1-3 times per week, or 2: ≧4 times per week or almost every day)

3. Discretion in work (0: yes or 1: no)

4. Average sleeping duration (0: <6 h, 1:6 to <8 h, or 2: ≧8 h)

5. Employment status (0: full-time employees [non-managerial]; 1: full-time employees [management] and company executives; 2: self-employed, freelance, and self-employed assistance; or 3: other)

6. Mental health (whether there has been at least one day in the past 30 days when your mental health has not been good) (0: no or 1: yes)

7. Physical activity (whether you walk or engage in equivalent physical activity in your daily life for at least 1 hour per day) (0: yes or 1: no)

8. Work type (0: desk worker, 1: worker talking to people, 2: physical worker).

To determine the characteristics of the study participants, summary statistics of the collected variables were calculated for each category defined by sedentary time (Analysis 1). For continuous variables, the median and first and third quartiles were calculated; for categorical variables, frequencies and percentages were calculated.

Then, a multivariate analysis (Analysis 2) was conducted with the independent variable being a categorical variable created based on the aforementioned sedentary time, and the dependent variable being a categorical variable created by defining "yes" and "somewhat yes" as high work engagement and other responses as low work engagement from the aforementioned categorical variable representing the degree of work engagement. Moreover, to evaluate the validity of the results of the aforementioned multivariate analysis, a sensitivity analysis (Analysis 3) was performed after changing the cutoff points to generate outcome variables. Specifically, the outcome variable was created by defining "yes" as high work engagement and other responses as low work engagement from the aforementioned categorical variable representing the level of work engagement.

Finally, to investigate whether the association between sedentary behavior and low work engagement differs for specific groups of desk workers, we conducted a subgroup analysis (Analyses 4 and 5). Specifically, we created subgroups of desk workers based on employment status and conducted multivariate analyses with the same independent and dependent variables as in Analysis 2. Another subgroup was created based on the frequency of working from home, and the same analysis was conducted.

Logistic regression analysis was used in Analysis 2 to Analysis 5, with a two-sided significance level of 5%. The R statistical software (version 4.3.0; R Foundation for Statistical Computing, Vienna, Austria) was used for all analyses.

## Results

Of the 33,000 participants, 32,657 were aged 18 years or older, of whom 21,327 were employed in jobs except domestic work. In addition, 600 of them were excluded who were considered to have outliers. As a result, 20,727 participants were included in the final analysis, of whom 11,687 were male (56%) and the median age was 42 years. Table 1 shows the baseline characteristics of the participants in the analysis, with 37.1% of participants reporting <4 h of sedentary time per day, 35.4% reporting 4 to <8 h, 18.3% reporting 8 to <12 h, and 9.2% reporting ≥12 h or unknown. For the main outcome of work engagement, 7.4% of participants answered "Yes," 39.7% answered "Somewhat yes," 34.3% answered "Somewhat no," and 18.6% answered "No" to the question, "Do you feel more energized when you work?”. In an analysis that included all work types, sedentary time was significantly associated with low work engagement, with a trend indicating that longer sedentary time was associated with lower work engagement (sedentary time, compared to the reference category “<4 hours/day”, 4 to <8 h: OR 1.26, 95% CI: 1.16-1.35; 8 to <12 h: OR 1.52, 95% CI: 1.39-1.67; ≥12 h or unknown: OR 1.68, 95% CI: 1.51-1.88, respectively) (Table 2). In addition, this association was stronger among desk workers (sedentary time, compared to the reference category “<4 hours/day”, 4 to <8 h: OR 1.42, 95% CI: 1.25-1.60; 8 to <12 h: OR 1.77, 95% CI: 1.55-2.01; ≥12 h or unknown: OR 2.14, 95% CI: 1.80-2.51, respectively) (Table 2). In addition, the sensitivity analysis showed similar results to the multivariate analysis described above (In the analysis including all work types, sedentary time, compared to the reference category “<4 hours/day”, 4 to <8 h: OR 1.3, 95% CI: 1.14-1.48; 8 to <12 h: OR 1.3, 95% CI: 1.09-1.54; ≥12 h: OR 1.46, 95% CI: 1.19-1.82, and in the analysis limited to desk workers, sedentary time, compared to the reference category “<4 hours/day”, 4 to <8 h: OR 1.68, 95% CI: 1.35-2.10; 8 to <12 h: OR 1.77, 95% CI: 1.39-2.23; ≥12 h: OR 2.41, 95% CI: 1.73-3.32) (Table 3).

**Table 1:** Characteristics of study participant (Analysis 1) ^a^n (%); median (Q1, Q3).

Variables	Total (n = 20,727^a^)	<4 h (n = 7,681^a^)	4 to <8 h (n = 7,345^a^)	8 to <12 h (n = 3,796^a^)	≧12 h or unknown (n = 1,905^a^)
Work engagement (whether you feel energized when you work)	-	-	-	-	-
Yes	1,532 (7.4%)	668 (8.7%)	506 (6.9%)	243 (6.4%)	115 (6.0%)
Somewhat yes	8,227 (40%)	3,290 (43%)	3,016 (41%)	1,312 (35%)	609 (32%)
Somewhat no	7,115 (34%)	2,531 (33%)	2,568 (35%)	1,362 (36%)	654 (34%)
No	3,853 (19%)	1,192 (16%)	1,255 (17%)	879 (23%)	527 (28%)
Gender	-	-	-	-	-
Male	11,687 (56%)	4,386 (57%)	4,116 (56%)	2,105 (55%)	1,080 (57%)
Female	9,040 (44%)	3,295 (43%)	3,229 (44%)	1,691 (45%)	825 (43%)
Age (years) (median(Q1, Q3))	42 (30, 55)	40 (29, 55)	45 (31, 58)	41 (29, 52)	41 (29, 51)
BMI (median (Q1, Q3))	21.5 (19.5, 23.7)	21.5 (19.5, 23.7)	21.5 (19.6, 23.8)	21.3 (19.4, 23.5)	21.3 (19.3, 23.9)
Most recent educational background	-	-	-	-	-
Middle and high school	4,831 (23%)	2,175 (28%)	1,582 (22%)	616 (16%)	458 (24%)
University	14,483 (70%)	5,164 (67%)	5,247 (71%)	2,793 (74%)	1,279 (67%)
Graduate school or Other	1,413 (6.8%)	342 (4.5%)	516 (7.0%)	387 (10%)	168 (8.8%)
Annual household income	-	-	-	-	-
<300 JPY	2,574 (12%)	1,108 (14%)	820 (11%)	411 (11%)	235 (12%)
300 to <800 JPY	9,442 (46%)	3,685 (48%)	3,348 (46%)	1,717 (45%)	692 (36%)
≧800 JPY	8,711 (42%)	2,888 (38%)	3,177 (43%)	1,668 (44%)	978 (51%)
Labor time per week	-	-	-	-	-
<40 h	7,835 (38%)	3,244 (42%)	2,957 (40%)	974 (26%)	660 (35%)
≧40 h	12,892 (62%)	4,437 (58%)	4,388 (60%)	2,822 (74%)	1,245 (65%)
Frequency of working from home	-	-	-	-	-
0-3 times per month	16,771 (81%)	6,727 (88%)	5,989 (82%)	2,637 (69%)	1,418 (74%)
1-3 times per week	2,098 (10%)	581 (7.6%)	776 (11%)	568 (15%)	173 (9.1%)
≧4 times per week or almost everyday	1,858 (9.0%)	373 (4.9%)	580 (7.9%)	591 (16%)	314 (16%)
Discretion in work	-	-	-	-	-
Yes	12,252 (59%)	4,050 (53%)	4,671 (64%)	2,477 (65%)	1,054 (55%)
No	8,475 (41%)	3,631 (47%)	2,674 (36%)	1,319 (35%)	851 (45%)
Average sleeping duration per day	-	-	-	-	-
<6 h	5,783 (28%)	2,587 (34%)	1,796 (24%)	944 (25%)	456 (24%)
6 to <8 h	11,932 (58%)	4,093 (53%)	4,665 (64%)	2,361 (62%)	813 (43%)
≧8 h	3,012 (15%)	1,001 (13%)	884 (12%)	491 (13%)	636 (33%)
Employment status	-	-	-	-	-
Full-time employees (non-managerial)	9,724 (47%)	3,348 (44%)	3,312 (45%)	2,166 (57%)	898 (47%)
Full-time employees (management) and company executives	3,470 (17%)	1,125 (15%)	1,367 (19%)	699 (18%)	279 (15%)
Self-employed, freelance, and self-employed assistance	2,078 (10%)	839 (11%)	739 (10%)	281 (7.4%)	219 (11%)
Other	5,455 (26%)	2,369 (31%)	1,927 (26%)	650 (17%)	509 (27%)
Physical activity (whether you walk or engage in equivalent physical activity in your daily life for at least 1 hour per day)	-	-	-	-	-
Yes	6,989 (34%)	2,725 (35%)	2,677 (36%)	1,168 (31%)	419 (22%)
No	13,738 (66%)	4,956 (65%)	4,668 (64%)	2,628 (69%)	1,486 (78%)
Mental health (whether there has been at least one day in the past 30 days when your mental health has not been good)	-	-	-	-	-
No	14,393 (69%)	5,482 (71%)	5,096 (69%)	2,486 (65%)	1,329 (70%)
Yes	6,334 (31%)	2,199 (29%)	2,249 (31%)	1,310 (35%)	576 (30%)
Work type	-	-	-	-	-
Desk worker	10,052 (48%)	1,606 (21%)	4,203 (57%)	3,106 (82%)	1,137 (60%)
Worker talking to people	4,881 (24%)	2,462 (32%)	1,686 (23%)	433 (11%)	300 (16%)
Physical worker	5,794 (28%)	3,613 (47%)	1,456 (20%)	257 (6.8%)	468 (25%)

**Table 2:** Results of a multivariate analysis examining the association between sedentary time per day and low work engagement after adjusting for several control variables (Analysis 2) ^a^The outcome was low work engagement, and low work engagement was defined as those who answered "somewhat no" or "No" to the question "Do you feel more energized when you work?".

Variables	All				Work type										
					Desk worker				Worker talking to people				Physical worker		
	OR^a^	95% CI	p-Value		OR^a^	95% CI	p-Value		OR^a^	95% CI	p-Value		OR^a^	95% CI	p-Value
Average sedentary behavior per day	-	-	-	-	-	-	-	-	-	-	-	-	-	-	-
<4 h	Ref	-	-	-	Ref	-	-	-	Ref	-	-	-	Ref	-	-
4 to <8 h	1.26	1.16, 1.35	<0.001	-	1.42	1.25, 1.6	<0.001	-	1.23	1.08, 1.4	0.002	-	1.15	1.01, 1.31	0.035
8 to <12 h	1.52	1.39, 1.67	<0.001	-	1.77	1.55, 2.01	<0.001	-	1.3	1.04, 1.62	0.018	-	1.35	1.04, 1.77	0.027
≧12 h or unknown	1.68	1.51, 1.88	<0.001	-	2.14	1.8, 2.51	<0.001	-	1.6	1.23, 2.08	<0.001	-	1.23	1, 1.54	0.049
Gender	-	-	-	-	-	-	-	-	-	-	-	-	-	-	-
Male	Ref	-	-	-	Ref	-	-	-	Ref	-	-	-	Ref	-	-
Female	0.85	0.79, 0.9	<0.001	-	0.99	0.9, 1.09	0.8	-	0.79	0.69, 0.91	0.002	-	0.7	0.63, 0.8	<0.001
BMI	1	0.99, 1.01	>0.9	-	1	0.99, 1.02	0.8	-	1	0.98, 1.02	0.9	-	1	0.98, 1.02	0.9
Age (years)	1	0.99, 1	<0.001	-	0.99	0.99, 1	<0.001	-	1	0.99, 1	0.035	-	1	0.99, 1	0.3
Most recent educational background	-	-	-	-	-	-	-	-	-	-	-	-	-	-	-
Middle and high school	Ref	-	-	-	Ref	-	-	-	Ref	-	-	-	Ref	-	-
University	0.87	0.81, 0.93	<0.001	-	0.95	0.84, 1.06	0.4	-	0.84	0.72, 0.97	0.019	-	0.84	0.74, 0.93	0.002
Graduate school or other	0.7	0.61, 0.79	<0.001	-	0.71	0.59, 0.84	<0.001	-	0.78	0.57, 1.06	0.12	-	0.9	0.67, 1.2	0.5
Annual household income	-	-	-	-	-	-	-	-	-	-	-	-	-	-	-
<300 JPY	Ref	-	-	-	Ref	-	-	-	Ref	-	-	-	Ref	-	-
300 to <800 JPY	0.81	0.74, 0.9	<0.001	-	0.83	0.71, 0.96	0.015	-	0.89	0.73, 1.07	0.2	-	0.76	0.64, 0.89	<0.001
≧800 JPY	0.79	0.71, 0.86	<0.001	-	0.79	0.68, 0.91	0.002	-	0.84	0.7, 1.03	0.1	-	0.76	0.64, 0.89	<0.001
Labor time per week	-	-	-	-	-	-	-	-	-	-	-	-	-	-	-
<40 h	Ref	-	-	-	Ref	-	-	-	Ref	-	-	-	Ref	-	-
≧40 h	1.14	1.06, 1.22	<0.001	-	1.02	0.92, 1.14	0.6	-	1.27	1.09, 1.48	0.002	-	1.22	1.06, 1.39	0.004
Frequency of working from home	-	-	-	-	-	-	-	-	-	-	-	-	-	-	-
0-3 times per month	Ref	-	-	-	Ref	-	-	-	Ref	-	-	-	Ref	-	-
1-3 times per week	0.89	0.8, 0.98	0.017	-	0.86	0.76, 0.97	0.012	-	1.04	0.84, 1.28	0.7	-	0.76	0.55, 1.07	0.12
≧4 times per week or almost every day	0.99	0.89, 1.11	0.8	-	0.9	0.79, 1.03	0.15	-	1.22	0.9, 1.63	0.2	-	1.27	0.9, 1.82	0.2
Discretion in work	-	-	-	-	-	-	-	-	-	-	-	-	-	-	-
Yes	Ref	-	-	-	Ref	-	-	-	Ref	-	-	-	Ref	-	-
No	2.27	2.14, 2.41	<0.001	-	2.39	2.18, 2.61	<0.001	-	2.39	2.12, 2.69	<0.001	-	2.18	1.95, 2.44	<0.001
Average sleeping duration per day	-	-	-	-	-	-	-	-	-	-	-	-	-	-	-
<6 h	Ref	-	-	-	Ref	-	-	-	Ref	-	-	-	Ref	-	-
6 to <8 h	0.88	0.83, 0.94	<0.001	-	0.94	0.85, 1.04	0.3	-	0.85	0.74, 0.98	0.022	-	0.8	0.7, 0.9	<0.001
≧8 h	0.84	0.76, 0.92	<0.001	-	0.83	0.72, 0.95	0.007	-	0.77	0.64, 0.94	0.01	-	0.94	0.79, 1.12	0.5
Employment status	-	-	-	-	-	-	-	-	-	-	-	-	-	-	-
Full-time employees (non-managerial)	Ref	-	-	-	Ref	-	-	-	Ref	-	-	-	Ref	-	-
Full-time employees (management) and company executives	0.7	0.64, 0.76	<0.001	-	0.73	0.66, 0.82	<0.001	-	0.69	0.58, 0.83	<0.001	-	0.7	0.58, 0.87	<0.001
Self-employed, freelance, and self-employed assistance	0.55	0.49, 0.62	<0.001	-	0.59	0.5, 0.7	<0.001	-	0.49	0.39, 0.61	<0.001	-	0.56	0.45, 0.7	<0.001
Other	0.95	0.87, 1.03	0.2	-	0.95	0.84, 1.08	0.4	-	0.9	0.75, 1.06	0.2	-	1.05	0.9, 1.22	0.5
Physical activity	-	-	-	-	-	-	-	-	-	-	-	-	-	-	-
Yes	Ref	-	-	-	Ref	-	-	-	Ref	-	-	-	Ref	-	-
No	1.35	1.27, 1.43	<0.001	-	1.31	1.2, 1.43	<0.001	-	1.39	1.22, 1.57	<0.001	-	1.38	1.22, 1.54	<0.001
Mental health	-	-	-	-	-	-	-	-	-	-	-	-	-	-	-
No	Ref	-	-	-	Ref	-	-	-	Ref	-	-	-	Ref	-	-
Yes	1.73	1.62, 1.84	<0.001	-	1.68	1.54, 1.86	<0.001	-	1.79	1.57, 2.03	<0.001	-	1.79	1.58, 2.03	<0.001
Work type	-	-	-	-	-	-	-	-	-	-	-	-	-	-	-
Desk worker	Ref	-	-	-	-	-	-	-	-	-	-	-	-	-	-
Worker talking to people	0.73	0.68, 0.79	<0.001	-	-	-	-	-	-	-	-	-	-	-	-
Physical worker	0.85	0.79, 0.92	<0.001	-	-	-	-	-	-	-	-	-	-	-	-

**Table 3:** Results of a sensitivity analysis investigating the association between sedentary time per day and low work engagement with the outcome variable changed from that in Analysis 2 (Analysis 3) ^a^The outcome was low work engagement, and low work engagement was defined as those who answered "Somewhat yes," "Somewhat no," or "No" to the question "Do you feel more energized when you work?".

Variables	All				Work type										
					Desk worker				Worker talking to people				Physical worker		
	OR^a^	95% CI	p-Value		OR^a^	95% CI	p-Value		OR^a^	95% CI	p-Value		OR^a^	95% CI	p-Value
Average sedentary behavior per day	-	-	-	-	-	-	-	-	-	-	-	-	-	-	-
<4 h	Ref	-	-	-	Ref	-	-	-	Ref	-	-	-	Ref	-	-
4 to <8 h	1.3	1.14, 1.48	<0.001	-	1.68	1.35, 2.1	<0.001	-	1.2	0.96, 1.51	0.11	-	1.19	0.93, 1.51	0.2
8 to <12 h	1.3	1.09, 1.54	0.004	-	1.77	1.39, 2.23	<0.001	-	1.19	0.82, 1.77	0.4	-	0.91	0.59, 1.46	0.7
≧12 h or unknown	1.46	1.19, 1.82	<0.001	-	2.41	1.73, 3.32	<0.001	-	1.08	0.71, 1.72	0.7	-	1.06	0.73, 1.57	0.8
Gender	-	-	-	-	-	-	-	-	-	-	-	-	-	-	-
Male	Ref	-	-	-	Ref	-	-	-	Ref	-	-	-	Ref	-	-
Female	0.84	0.73, 0.94	0.004	-	0.99	0.82, 1.2	>0.9	-	0.74	0.58, 0.95	0.016	-	0.7	0.55, 0.88	0.002
BMI	1.01	0.99, 1.03	0.3	-	1.03	1, 1.05	0.076	-	0.98	0.95, 1.02	0.4	-	1.01	0.98, 1.05	0.5
Age (years)	1.01	1.01, 1.01	<0.001	-	1	1, 1.01	0.2	-	1.01	1, 1.02	0.003	-	1.01	1.01, 1.02	<0.001
Most recent educational background	-	-	-	-	-	-	-	-	-	-	-	-	-	-	-
Middle and high school	Ref	-	-	-	Ref	-	-	-	Ref	-	-	-	Ref	-	-
University	0.98	0.86, 1.12	0.8	-	1	0.8, 1.25	>0.9	-	0.8	0.62, 1.04	0.11	-	1.11	0.9, 1.38	0.4
Graduate school or other	0.69	0.55, 0.86	<0.001	-	0.77	0.57, 1.05	0.1	-	0.53	0.33, 0.86	0.009	-	0.74	0.47, 1.2	0.2
Annual household income	-	-	-	-	-	-	-	-	-	-	-	-	-	-	-
<300 JPY	Ref	-	-	-	Ref	-	-	-	Ref	-	-	-	Ref	-	-
300 to <800 JPY	1.06	0.89, 1.26	0.5	-	1.14	0.84, 1.51	0.4	-	1.14	0.82, 1.55	0.4	-	0.94	0.7, 1.27	0.7
≧800 JPY	0.94	0.79, 1.12	0.5	-	1.06	0.79, 1.4	0.7	-	1.07	0.78, 1.46	0.7	-	0.76	0.55, 1.01	0.064
Labor time per week	-	-	-	-	-	-	-	-	-	-	-	-	-	-	-
<40 h	Ref	-	-	-	Ref	-	-	-	Ref	-	-	-	Ref	-	-
≧40 h	1.11	0.97, 1.26	0.13	-	0.99	0.81, 1.2	>0.9	-	1.02	0.79, 1.3	>0.9	-	1.28	1.01, 1.65	0.039
Frequency of working from home	-	-	-	-	-	-	-	-	-	-	-	-	-	-	-
0-3 times per month	Ref	-	-	-	Ref	-	-	-	Ref	-	-	-	Ref	-	-
1-3 times per week	0.95	0.8, 1.14	0.6	-	0.9	0.73, 1.15	0.4	-	1.04	0.74, 1.49	0.8	-	1.02	0.6, 1.86	>0.9
≧4 times per week or almost every day	0.86	0.72, 1.04	0.11	-	0.79	0.63, 1.01	0.058	-	0.74	0.5, 1.11	0.13	-	1.05	0.63, 1.84	0.9
Discretion in work	-	-	-	-	-	-	-	-	-	-	-	-	-	-	-
Yes	Ref	-	-	-	Ref	-	-	-	Ref	-	-	-	Ref	-	-
No	2.34	2.05, 2.66	<0.001	-	2.12	1.72, 2.61	<0.001	-	2.16	1.72, 2.72	<0.001	-	3	2.36, 3.67	<0.001
Average sleeping duration per day	-	-	-	-	-	-	-	-	-	-	-	-	-	-	-
<6 h	Ref	-	-	-	Ref	-	-	-	Ref	-	-	-	Ref	-	-
6 to <8 h	1.03	0.91, 1.17	0.6	-	1	0.82, 1.21	>0.9	-	1.04	0.82, 1.32	0.7	-	1	0.79, 1.26	>0.9
≧8 h	0.84	0.72, 1	0.051	-	0.72	0.56, 0.93	0.011	-	0.86	0.63, 1.19	0.4	-	1.04	0.76, 1.45	0.8
Employment status	-	-	-	-	-	-	-	-	-	-	-	-	-	-	-
Full-time employees (non-managerial)	Ref	-	-	-	Ref	-	-	-	Ref	-	-	-	Ref	-	-
Full-time employees (management) and company executives	0.61	0.52, 0.71	<0.001	-	0.72	0.58, 0.9	0.003	-	0.54	0.4, 0.73	<0.001	-	0.6	0.42, 0.88	0.007
Self-employed, freelance, and self-employed assistance	0.39	0.33, 0.46	<0.001	-	0.54	0.41, 0.73	<0.001	-	0.3	0.22, 0.44	<0.001	-	0.3	0.22, 0.43	<0.001
Other	0.87	0.73, 1.02	0.087	-	1.15	0.88, 1.52	0.3	-	0.68	0.49, 0.93	0.017	-	0.83	0.61, 1.11	0.2
Physical activity	-	-	-	-	-	-	-	-	-	-	-	-	-	-	-
Yes	Ref	-	-	-	Ref	-	-	-	Ref	-	-	-	Ref	-	-
No	1.42	1.27, 1.58	<0.001	-	1.48	1.25, 1.73	<0.001	-	1.45	1.17, 1.79	<0.001	-	1.36	1.11, 1.67	0.004
Mental health	-	-	-	-	-	-	-	-	-	-	-	-	-	-	-
No	Ref	-	-	-	Ref	-	-	-	Ref	-	-	-	Ref	-	-
Yes	1.7	1.49, 1.93	<0.001	-	1.68	1.38, 2.05	<0.001	-	1.7	1.34, 2.18	<0.001	-	1.72	1.35, 2.23	<0.001
Work type	-	-	-	-	-	-	-	-	-	-	-	-	-	-	-
Desk worker	Ref	-	-	-	-	-	-	-	-	-	-	-	-	-	-
Worker talking to people	0.74	0.64, 0.85	<0.001	-	-	-	-	-	-	-	-	-	-	-	-
Physical worker	0.79	0.68, 0.92	0.003	-	-	-	-	-	-	-	-	-	-	-	-

The association between sedentary time and low work engagement was stronger in the groups of full-time workers who were non-managers and those who worked from home ≥4 days weekly (in the group of full-time workers who were non-managers, sedentary time, compared to the reference category “<4 hours/day”, 4 to <8 h: OR 2.14, 95% CI: 1.52-3.00; 8 to <12 h: OR 2.10, 95% CI: 1.46-3.00; ≥12 h or unknown: OR 3.32, 95% CI: 1.99-6.05; in those with work from home frequency of ≥4 days weekly, sedentary time, compared to the reference category “<4 hours/day”, 4 to <8 h: OR 1.46, 95% CI: 0.99-2.16; 8 to <12 h: OR 1.73, 95% CI: 1.19-2.56; ≥12 h or unknown: OR 2.41, 95% CI: 1.58-3.67) (Table 4, Table 5). 

**Table 4:** Result of a multivariate analysis in subgroups constructed by employment status investigating the association between sedentary time per day and low work engagement (Analysis 4) ^a^The outcome was low work engagement, and low work engagement was defined as those who answered "somewhat no" or "No" to the question "Do you feel more energized when you work?".

Variables	Employment status														
	Full-time employees (non-managerial)				Full-time employees (management) and company executives"				Self-employed, freelance, and self-employed assistance				Other		
	OR^a^	95% CI	p-Value		OR^a^	95% CI	p-Value		OR^a^	95% CI	p-Value		OR^a^	95% CI	p-Value
Average sedentary behavior per day	-	-	-	-	-	-	-	-	-	-	-	-	-	-	-
<4 h	Ref	-	-	-	Ref	-	-	-	Ref	-	-	-	Ref	-	-
4 to <8 h	2.14	1.52, 3	<0.001	-	1.75	1.14, 2.66	0.01	-	0.92	0.52, 1.6	0.8	-	1.54	0.9, 2.56	0.11
8 to <12 h	2.1	1.46, 3	<0.001	-	1.95	1.22, 3	0.005	-	0.75	0.39, 1.4	0.4	-	2.27	1.16, 4.48	0.018
≧12 h or unknown	3.32	1.99, 6.05	<0.001	-	1.86	1, 3.67	0.058	-	1.21	0.54, 2.72	0.7	-	2.72	1.13, 7.39	0.036
Gender	-	-	-	-	-	-	-	-	-	-	-	-	-	-	-
Male	Ref	-	-	-	Ref	-	-	-	Ref	-	-	-	Ref	-	-
Female	1.07	0.82, 1.42	0.6	-	0.84	0.57, 1.3	0.4	-	0.93	0.59, 1.49	0.8	-	0.77	0.45, 1.3	0.3
BMI	1.04	1, 1.08	0.083	-	1.04	0.98, 1.11	0.2	-	1.03	0.96, 1.11	0.4	-	0.96	0.9, 1.03	0.3
Age (years)	1.01	1, 1.02	0.2	-	1	0.99, 1.02	0.6	-	1	0.99, 1.02	0.6	-	1	0.98, 1.01	0.8
Most recent educational background	-	-	-	-	-	-	-	-	-	-	-	-	-	-	-
Middle and high school	Ref	-	-	-	Ref	-	-	-	Ref	-	-	-	Ref	-	-
University	0.84	0.56, 1.25	0.4	-	1.32	0.85, 1.99	0.2	-	0.73	0.41, 1.26	0.3	-	1.3	0.8, 2.05	0.3
Graduate school or other	0.68	0.41, 1.12	0.13	-	1.32	0.73, 2.48	0.4	-	0.4	0.18, 0.88	0.021	-	0.79	0.33, 2.27	0.6
Annual household income	-	-	-	-	-	-	-	-	-	-	-	-	-	-	-
<300 JPY	Ref	-	-	-	Ref	-	-	-	Ref	-	-	-	Ref	-	-
300 to <800 JPY	0.74	0.42, 1.23	0.3	-	1.67	0.73, 3.32	0.2	-	1.72	0.94, 3	0.072	-	1.08	0.59, 1.9	0.8
≧800 JPY	0.76	0.42, 1.26	0.3	-	1.25	0.55, 2.53	0.6	-	1.43	0.82, 2.46	0.2	-	1.04	0.57, 1.86	0.9
Labor time per week	-	-	-	-	-	-	-	-	-	-	-	-	-	-	-
<40 h	Ref	-	-	-	Ref	-	-	-	Ref	-	-	-	Ref	-	-
≧40 h	0.99	0.72, 1.34	>0.9	-	0.84	0.54, 1.27	0.4	-	0.98	0.62, 1.57	>0.9	-	1.25	0.74, 2.14	0.4
Frequency of working from home	-	-	-	-	-	-	-	-	-	-	-	-	-	-	-
0-3 times per month	Ref	-	-	-	Ref	-	-	-	Ref	-	-	-	Ref	-	-
1-3 times per week	0.89	0.64, 1.26	0.5	-	0.92	0.62, 1.42	0.7	-	0.67	0.37, 1.28	0.2	-	1.57	0.71, 4.06	0.3
≧4 times per week or almost every day	0.84	0.58, 1.25	0.4	-	0.73	0.46, 1.21	0.2	-	0.9	0.53, 1.49	0.7	-	0.55	0.3, 1.13	0.084
Discretion in work	-	-	-	-	-	-	-	-	-	-	-	-	-	-	-
Yes	Ref	-	-	-	Ref	-	-	-	Ref	-	-	-	Ref	-	-
No	2.48	1.84, 3.32	<0.001	-	1.7	1.16, 2.53	0.008	-	2.64	1.23, 6.69	0.021	-	1.82	1.09, 3.32	0.026
Average sleeping duration per day	-	-	-	-	-	-	-	-	-	-	-	-	-	-	-
<6 h	Ref	-	-	-	Ref	-	-	-	Ref	-	-	-	Ref	-	-
6 to <8 h	1.19	0.88, 1.58	0.3	-	0.9	0.62, 1.31	0.6	-	0.81	0.46, 1.39	0.5	-	0.88	0.49, 1.49	0.6
≧8 h	0.9	0.61, 1.34	0.6	-	0.68	0.41, 1.16	0.2	-	0.65	0.33, 1.26	0.2	-	0.46	0.25, 0.9	0.024
Physical activity	-	-	-	-	-	-	-	-	-	-	-	-	-	-	-
Yes	Ref	-	-	-	Ref	-	-	-	Ref	-	-	-	Ref	-	-
No	1.42	1.09, 1.84	0.009	-	1.55	1.11, 2.16	0.01	-	1.45	0.93, 2.23	0.1	-	1.55	1, 2.41	0.049
Mental health	-	-	-	-	-	-	-	-	-	-	-	-	-	-	-
No	Ref	-	-	-	Ref	-	-	-	Ref	-	-	-	Ref	-	-
Yes	1.4	1.06, 1.88	0.018	-	1.92	1.26, 3	0.004	-	2.2	1.31, 4.06	0.004	-	1.84	1.08, 3.32	0.031

**Table 5:** Result of a multivariate analysis in subgroups constructed by frequency of working from home investigating the association between sedentary time per day and low work engagement (Analysis 5) ^a^The outcome was low work engagement, and low work engagement was defined as those who answered "somewhat no" or "No" to the question "Do you feel more energized when you work?".

Variables	Frequency of working from home										
	0-3 times per month				1-3 times per week				≧4 times per week or almost every day		
	OR^a^	95% CI	p-Value		OR^a^	95% CI	p-Value		OR^a^	95% CI	p-Value
Average sedentary behavior per day	-	-	-	-	-	-	-	-	-	-	-
<4 h	-	-	-	-	-	-	-	-	-	-	-
4 to <8 h	1.39	1.2, 1.6	<0.001	-	1.48	1.07, 2.03	0.017	-	1.46	0.99, 2.16	0.055
8 to <12 h	1.79	1.52, 2.1	<0.001	-	1.68	1.21, 2.36	0.002	-	1.73	1.19, 2.56	0.005
≧12 h or unknown	2.16	1.75, 2.64	<0.001	-	1.58	1.02, 2.46	0.039	-	2.41	1.58, 3.67	<0.001
Gender	-	-	-	-	-	-	-	-	-	-	-
Male	-	-	-	-	-	-	-	-	-	-	-
Female	1.04	0.92, 1.17	0.5	-	0.93	0.72, 1.2	0.6	-	0.85	0.66, 1.11	0.2
BMI	1	0.99, 1.02	0.6	-	0.97	0.93, 1.01	0.12	-	1.01	0.97, 1.05	0.6
Age (years)	0.99	0.99, 1	0.002	-	1	0.99, 1.01	0.4	-	0.98	0.98, 0.99	<0.001
Last educational background	-	-	-	-	-	-	-	-	-	-	-
Middle and high school	-	-	-	-	-	-	-	-	-	-	-
University	0.95	0.84, 1.07	0.4	-	1.26	0.85, 1.88	0.2	-	0.83	0.59, 1.15	0.3
Graduate school or other	0.72	0.58, 0.89	0.002	-	0.9	0.56, 1.46	0.7	-	0.62	0.39, 0.96	0.032
Annual household income	-	-	-	-	-	-	-	-	-	-	-
<300 JPY	-	-	-	-	-	-	-	-	-	-	-
300 to <800 JPY	0.93	0.78, 1.11	0.4	-	0.66	0.41, 1.06	0.088	-	0.57	0.39, 0.83	0.003
≧800 JPY	0.82	0.68, 0.98	0.026	-	0.77	0.48, 1.23	0.3	-	0.64	0.44, 0.93	0.021
Labor time per week	-	-	-	-	-	-	-	-	-	-	-
<40 h	-	-	-	-	-	-	-	-	-	-	-
≧40 h	0.99	0.88, 1.12	0.9	-	1.26	0.96, 1.65	0.09	-	0.93	0.71, 1.21	0.6
Discretion in work	-	-	-	-	-	-	-	-	-	-	-
Yes	-	-	-	-	-	-	-	-	-	-	-
No	2.25	2.01, 2.48	<0.001	-	3	2.41, 4.06	<0.001	-	2.72	2.12, 3.67	<0.001
Average sleeping duration per day	-	-	-	-	-	-	-	-	-	-	-
<6 h	-	-	-	-	-	-	-	-	-	-	-
6 to <8 h	0.95	0.84, 1.06	0.4	-	0.96	0.74, 1.25	0.8	-	0.88	0.65, 1.17	0.4
≧8 h	0.86	0.73, 1.03	0.1	-	0.79	0.57, 1.13	0.2	-	0.71	0.5, 1.02	0.062
Employment status	-	-	-	-	-	-	-	-	-	-	-
Full-time employees (non-managerial)	-	-	-	-	-	-	-	-	-	-	-
Full-time employees (management) and company executives	0.75	0.65, 0.85	<0.001	-	0.73	0.55, 0.96	0.027	-	0.62	0.44, 0.88	0.007
Self-employed, freelance, and self-employed assistance	0.57	0.43, 0.76	<0.001	-	0.66	0.43, 1.03	0.07	-	0.54	0.4, 0.75	<0.001
Other	0.89	0.77, 1.03	0.12	-	1.34	0.91, 1.95	0.14	-	1.01	0.68, 1.52	>0.9
Physical activity	-	-	-	-	-	-	-	-	-	-	-
Yes	-	-	-	-	-	-	-	-	-	-	-
No	1.32	1.19, 1.48	<0.001	-	1.34	1.05, 1.7	0.017	-	1.21	0.94, 1.55	0.14
Mental health	-	-	-	-	-	-	-	-	-	-	-
No	-	-	-	-	-	-	-	-	-	-	-
Yes	1.6	1.43, 1.8	<0.001	-	1.65	1.3, 2.1	<0.001	-	2.14	1.67, 2.72	<0.001

## Discussion

This study analyzed the association between sedentary time and work engagement among workers in Japan in the work environment after the COVID-19 pandemic. We found a significant association between sedentary time and low work engagement and observed that the association was particularly strong among desk workers. The results of this study are consistent with those of previous studies. Research on individuals born in 1966 indicated an association between sedentary time and levels of work engagement [5]. Furthermore, a study focusing on Japanese individuals aged 20-59 years revealed that those with extended periods of sedentary behavior were 1.49 times more likely to experience lower work engagement [6]. Moreover, a cross-sectional analysis highlighted an association between sedentary behavior and work engagement among office workers; this association remained significant even after accounting for occupational differences [7]. The sensitivity analysis showed a tendency similar to that of the previous studies mentioned above. Furthermore, in analyses by subgroups, where desk workers were categorized based on employment status and frequency of work from home, the strength of the association between sedentary behavior and low work engagement was more pronounced for those in full-time, non-managerial employment and those with work from home ≥4 days weekly. Thus, it was consistent with the results of previous studies that the strength of the association between sedentary time and low work engagement was not uniform across subgroups [6].

Unlike previous studies, this study covered various employment types, and the association between sedentary time and low work engagement tended to be the same for most employment types, except for the self-employed and freelance workers. We predict that the reason for this is that self-employed and freelance workers are more likely to interrupt sedentary time even when total sedentary time is long because the nature of their employment status allows them to interrupt their work whenever they want and to exercise relatively during work breaks, thus erasing the association between sedentary time and lower work engagement even when total sedentary time is long. Indeed, significant associations have been suggested between the frequency of interruptions in sedentary behavior and mood [26].

We believe that there are multiple mechanisms by which prolonged sedentary behavior affects work engagement. We considered the following mechanisms through which sedentary time affects work engagement. Specifically, prolonged sedentary behavior induces chronic pain, which increases discomfort at work, making it more difficult to work vigorously (i.e., low work engagement). Hergenroeder et al. suggested that prolonged sedentary behavior may influence the induction of chronic pain, and Malmberg-Ceder et al. showed that musculoskeletal pain may influence work engagement [27,28]. With regard to other mechanisms, the possibility that prolonged sedentary behavior is associated with productivity, thereby leading to a state of inability to work vividly, i.e. low work engagement, has also been raised [29]. However, further research on other mechanisms apart from those described above is warranted.

This study has two implications for occupational health. First, there is a need to measure sedentary behavior in a more validated way in the future and then conduct a prospective study to confirm this relationship. Second, to prevent low work engagement, strategies other than reducing sedentary time should be used in combination depending on the characteristics of the population. This is because the results of the subgroup analyses in this study suggest that among desk workers, the strength of the association between sedentary time and low work engagement varies according to group characteristics, for example, the association between the two is stronger for those who are full-time non-managerial employees and who work from home ≥4 days weekly. 

Our study has several limitations. First, this was a cross-sectional study; therefore, the possibility of causal inversion is undeniable. Therefore, in the future, it will be necessary to verify the causal relationship by conducting prospective studies or randomized controlled trials. Second, the sedentary time data used in the analysis were obtained from a self-response questionnaire. A previous study concluded that the correlation between self-reported sedentary time and objectively measured sedentary time was low [30]. Therefore, in the future, it will be necessary to objectively measure sedentary time, for example, using an accelerometer, to obtain highly valid data. Third, the sedentary time at work was not assessed. In the future, it will be necessary to take measures such as asking about sedentary time at work when preparing the questionnaire or wearing accelerometers to measure sedentary time only during work hours. The fourth limitation was the presence of unmeasured confounding factors. Future analyses that include possible new confounding variables in the model may yield new results.

## Conclusions

This study revealed a significant association between sedentary time and low work engagement among workers in Japan after the COVID-19 pandemic. The above associations were more pronounced among desk workers who were full-time non-managerial employees and those who worked at home ≥4 days weekly. In the future, prospective studies are needed to confirm this relationship using more validated measures of sedentary behavior.
